# A randomized control trial of phototherapy and 20% albumin versus phototherapy and saline in Kilifi, Kenya

**DOI:** 10.1186/s13104-019-4632-2

**Published:** 2019-09-23

**Authors:** Dorcas N. Magai, Michael Mwaniki, Amina Abubakar, Shebe Mohammed, Anne L. Gordon, Raphael Kalu, Paul Mwangi, Hans M. Koot, Charles R. Newton

**Affiliations:** 1 Centre for Geographic Medicine Research Coast, Kenya Medical Research Institute, Wellcome Trust Research Programme, PO Box 230-8010, Kilifi, Kenya; 2 Department of Clinical Neuro- and Developmental Psychology, Vrije Universiteit, Van der Boechorststraat 7, 1081 BT Amsterdam, The Netherlands; 3 Afya Research Africa, Nairobi, 00202 Kenya; 4 Department of Public Health, Pwani University, P.O. Box 195-80108, Kilifi, Kenya; 5 Institute for Human Development, Aga Khan University, P.O. Box 30270-00100, Nairobi, Kenya; 6 Department of Psychiatry, University of Oxford, Oxford, OX3 7JX UK; 7 Faculty of Life Sciences and Medicine, King’s College London, Strand, London, WC2R2LS England UK

**Keywords:** Albumin, Saline, Neonates, Neonatal jaundice, Sub-Saharan Africa

## Abstract

**Objective:**

The study evaluated the efficacy of phototherapy and 20% albumin infusion to reduce total serum bilirubin (TSB) in neonates with severe hyperbilirubinemia. The primary outcome was a reduction of TSB at the end of treatment. The secondary outcomes were the need for exchange transfusion, inpatient mortality, neurological outcomes at discharge, and development outcomes at 12-months follow-up.

**Results:**

One hundred and eighteen neonates were randomly assigned to phototherapy and 20% albumin (n = 59) and phototherapy and saline (n = 69). The median age at admission was 5 (interquartile range (IQR) 3–6) days, and the median gestation was 36 (IQR 36–38) weeks. No significant differences were found in the change in TSB (Mann–Whitney *U *=609, *p *= 0.98) and rate of change in TSB per hour after treatment (Mann–Whitney *U *= 540, *p *= 0.39) between the two groups. There were no significant differences between the two groups in the proportion of participants who required exchange transfusion (χ^2^ (2) = 0.36, p = 0.546); repeat phototherapy (χ^2^ (2) = 2.37, p = 0.123); and those who died (χ^2^ (2) = 0.92, p = 0.337). *Trial registration* The trial was registered in the International Standardized Randomized Controlled Trial Number (ISRCTN); trial registration number ISRCTN89732754.

## Introduction

Neonatal jaundice (NNJ) is a common condition [1] and occurs when the breakdown of red blood cells is increased leading to an accumulation of bilirubin [2]. Globally, the overall incidence of NNJ is 9.9 (2.8–35.6) per 10,000 live births [1]. Africa has the highest number of children developing NNJ with an incidence of 667.8 (603.4–738.5) per 10,000 live births [1]. In Kilifi County Hospital (KCH) Kenya, NNJ accounted for 24% of young infants’ mortality and was the primary diagnosis of 17% of over 1000 neonatal admissions during the first 60 days of life in 2003 [3]. Neonates may develop hyperbilirubinemia; total serum bilirubin (TSB) above the 95th percentile for that age (in hours). Phototherapy is the treatment of choice as it enhances the conversion of bilirubin into less toxic water-soluble photo-isomers [4] and is effective in the management of NNJ [5–9]. Severe unconjugated hyperbilirubinemia can cause neurotoxicity that leads to brain damage in neonates [10] and is associated with irreversible neurodevelopmental, and neurological impairment [11–16].

The use of blue-light fluorescent tubes during phototherapy increases the need for maintenance fluid by 25% and additional intravenous fluid 1–1.5 times the maintenance fluid during intensive phototherapy [17]. The increased fluid, mainly dextrose water is needed to compensate for fluid lost in the excretion of the water-soluble photo-products from bilirubin conversion. Lack of fluid may lead to dehydration in children undergoing phototherapy [18]. Moreover, adequate hydration and urine output improve the efficacy of phototherapy.

Albumin infusion during phototherapy is reported to induce a rapid decline in unconjugated bilirubin during phototherapy [19] since albumin binds to bilirubin. Hosono et al. reported that 25% albumin infusion was more effective in reducing serum levels of unbound bilirubin during the first 6 and 24 h of treatment compared to phototherapy alone [20], but did not significantly reduce TSB levels at the end of treatment. In another study, it was reported that 10% albumin had a protective effect against bilirubin toxicity and may be useful in children with severe hyperbilirubinemia [21]. Hosono et al. further report that albumin infusion therapy is associated with better hearing outcomes at 6 months compared to standard care [22].

Despite the knowledge that albumin infusion during phototherapy may be useful, most of the evidence is from high-income countries, and less is known in sub-Saharan Africa (SSA) despite the high incidence of NNJ. The present study evaluated the efficacy of phototherapy and 20% albumin infusion versus standard care (phototherapy and saline) in the reduction of bilirubin, exchange transfusion, inpatient mortality, neurological outcomes at discharge and development outcomes at 12-months.

## Main text

### Methods

#### Trial design

This is a randomized controlled trial of phototherapy and 20% albumin versus phototherapy and saline. The enrolled participants were randomized in a ratio of 1:1 to the albumin group and saline group.

#### Participants

The study was conducted at KCH from 2006 to 2011. All neonates admitted to KCH had a clinical assessment of NNJ. TSB was measured in neonates (0–30 days) admitted to the KCH paediatric ward on the day of admission. Those with a TSB above 250 μmol/L were investigated to determine the cause of hyperbilirubinemia and were randomized to either phototherapy and 20% albumin or phototherapy and saline. The decision to start phototherapy was calculated based on the postnatal age, gestational, and TSB levels at admission [23]. Phototherapy was initiated when TSB levels were from 85µ/mols/L/kg in babies below 2.5 kg (as a marker of prematurity). For sick neonates, treatment was started at 30µ/mols/L below the levels for the well baby. Participants were excluded from the study if they had a congenital abnormality, clinical evidence of kernicterus, or obstructive jaundice.

#### Interventions

Neonates randomized to receive albumin were given one infusion of intravenous 20% albumin (1 g/kg) over 2 h. The neonates that were randomized to 1/5 (0.9%) normal saline sodium 154 mmol/L and chloride 154 mmol/L in 10% dextrose received the same volume of fluid as those randomized to 20% albumin i.e. 5 ml/kg over a period of 2 h. Hyperbilirubinemia was managed using Photolight, Model AS20 220–230 V with YZ20BT132/20W blue fluorescent tubes. All children were on a routine antibiotic cover (Benzyl Penicillin and Gentamicin) and maintenance fluids (10% dextrose) [23]. Discharge from the hospital occured only when the TSB was below phototherapy level (less than 85/μmol/l/kg) and after 24 h observation to check for rebound hyperbilirubinemia after phototherapy was stopped.

Blood samples were taken for laboratory tests such as blood group determination and Coombs test (direct and indirect). Haemolytic causes were defined as mother-baby blood incompatibility or Coombs test positive. TSB was measured at admission, at 2 h, 6 h, 24 h, 48 h post admission, and every 24 h thereafter until the child was discharged. Participants were withdrawn from phototherapy if there was a need for exchange transfusion, hospital transfer, or death.

#### Study Outcomes

The primary study outcome was the reduction in TSB levels for the participants at the end of treatment. The secondary outcomes were the prevention of exchange transfusions during treatment, reduced inpatient mortality, neurological outcomes at discharge and developmental outcomes at 12-months.

#### Neurological and developmental assessment

A neurological assessments were performed using a clinical evaluation proforma designed for this study and assessed at discharge by a clinician blinded to the participants’ treatment arm. The developmental assessments were taken at 12-months of age by trained assessors. The Kilifi Development Inventory (KDI) [24, 25] and the Developmental Milestones Checklist (DMC) [24, 25] were administered to evaluate psychomotor, language and social emotional function.

#### Sample size

The sample size was based on previous data from KCH in which 48% of children with severe hyperbilirubinaemia had neurological deficits [11]. To detect a 50% difference in neurological sequelae in the albumin-treated group, assuming 80% power, 70 participants in both arms were required giving a total of 140 participants. Adjustment for death and loss to follow up of 10% required 80 participants in each arm giving a total of 160 participants.

#### Enrolment and randomization

The randomization was undertaken using card envelopes in blocks of 20. The random numbers were generated by the principal investigator and kept in serially numbered sealed opaque envelopes which were only opened after participants were enrolled in the study. However, due to the nature of this study, it was not possible for the investigators, assessors, clinicians, and parents to be blinded to the treatment arm once the participants were randomized.

#### Statistical analysis

Data were recorded on files, and double entered to a secure, Web-based REDCap database and analysed using STATA (version 15) [26]. Descriptive statistics such as frequencies, percentages, and median with respective interquartile ranges (IQR) were reported. Wilcoxon test was used to compare differences in TSB at admission and after treatment, while Mann–Whitney U test was used to compare the differences in change in TSB (the difference in TSB levels at the start and end of treatment) and rate of change per hour in TSB (the difference in the level of TSB at admission and at the end of treatment divided by the total number of hours taken on phototherapy) between the two treatments. Chi square test was used for comparisons of the proportion of participants who required exchange transfusion, repeat phototherapy, died, and participants who were discharged in the two treatments.

### Results

One hundred and fifty-six participants were randomized in the study (75 assigned to 20% albumin while 76 assigned to saline).

Demographic characteristics and proportion of participants who required exchange transfusion were analysed on 118 participants (59 in the 20% albumin arm and 69 in the saline arm).

Reduction in TSB levels in the two treatments was assessed on the 70 participants (34 in 20% albumin arm and 36 in the saline arm) whose data were available (see Additional file 1: Figure S1).

#### Participant characteristics

The baseline characteristics of participants enrolled in the study are given in Table 1. There were no significant differences in the demographic and baseline characteristics between the participants who received phototherapy and albumin and phototherapy and saline at baseline. The median age at admission was 5 (IQR 3–6) days, the median gestation was 36 (IQR 36–38) weeks, and the median age for the mothers was 24 (IQR 19–28) years. Most of the participants (40.8%) had haemolytic causes of jaundice, 14.6% had non-haemolytic causes, 15.4% had both haemolytic and non-haemolytic causes, while in 29.2% of the participants the causes of jaundice were unknown.

**Table 1:** Baseline characteristics participants

Characteristics	20% albumin + phototherapy (n = 59)	Saline + phototherapy (n = 69)	Test statistic
Age at admission (days)			
Median (IQR)	5 (3–6)	5 (4–6)	*P *=0.574*
Estimated gestational weeks			
Median (IQR)	36 (36–37)	36 (36–38)	–
Total serum bilirubin at admission			
Median (IQR)	368 (303–456)	392 (316–510)	*P *=0.331*
Mother’s age			
Median (IQR)	24 (19–29)	24 (20–27)	*P *=0.331*
ABO incompatibility *n*			
Yes	28	25	χ^2^(2) = 1.85, *P *=0.173
No	18	28	
Non-haemolytic causes *n*			
Yes	10	9	χ^2^(2) = 0.21, *P *=0.646
No	36	41	
Both haemolytic and non-haemolytic *n*			
Yes	5	3	χ^2^(2) = 0.92, *P *=0.336
No	54	66	
Unknown causes			
Yes	13	18	χ^2^(2) = 0.28, *P *=0.594
No	46	51	

* Mann U Whitney

#### Reduction in bilirubin levels after treatment

The participants’ TSB levels after treatment were significantly lower than TSB levels at admission in both the phototherapy and albumin (Z = 5.1, p < 0.00) and phototherapy and saline (Z = 5.2, p < 0.001) groups. There were no significant differences in the change in TSB (Mann–Whitney *U *=609, *p *= 0.98) and rate of change in TSB per hour (Mann–Whitney *U *= 540, *p *= 0.39) between the two groups (Table 2).

**Table 2:** Reduction in bilirubin levels at end of treatment in whom complete data were available

Variable	20% albumin + phototherapy (n = 34)	Saline + phototherapy (n = 36)
TSB at admission mean (SD)	387 (114)	370 (102)
TSB after phototherapy mean (SD)	191 (67)	188 (69)
Change in bilirubin*		
Median (IQR)	152 (101–248)	159 (101–230)
Rate of change (mg/dL/h)*		
Median (IQR)	2.16 (1.5–4.25)	1.91 (1.19–4.00)

Descriptive statistics

*SD* standard deviation

* No significant differences in change of total serum bilirubin and rate of change in total serum reduction per hour between the two arms of the trial p > 0.05

#### Outcome after treatment

There were no significant differences (*p *> 0.05) in the proportion of participants who required exchange transfusion (three in albumin group versus four in saline group), repeat phototherapy (two in albumin versus none in saline), those who died (six in albumin group versus eleven in saline group), and participants who were discharged in the two treatments (Table 3).

**Table 3:** Outcome after treatment

	20% albumin (n = 59)	Saline (n = 69)	Test statistic
Type of discharge n (%)			
Alive	53 (89.8)	58 (84.1)	χ^2^ (2) = 0.920, *P* = 0.337
Died	6 (10.2)	11 (15.9)	
Repeat phototherapy n (%)			
Yes	2 (3.4)	0 (0.0)	χ^2^ (2) = 2.376, *P* = 0.123
No	57 (96.6)	69 (100.0)	
Exchange transfusion n (%)			
Yes	3 (4.4)	4 (6.8)	χ^2^ (2) = 0.364, *P* = 0.546
No	55 (95.6)	66 (93.2)	
Abnormal neurological functioning	–	–	

#### Developmental outcomes at age 12-months

There was no significant difference in the 20% album group and saline group in the scores for psychomotor (M = 26.5, SD = 11.5, versus M = 20.1, SD = 13.8), t (55) = 1.9, *p* = 0.060; language (M = 7.3, SD = 4.3 versus M = 5.4, SD = 4.2), t (55) = 1.7, *p* = 0.090 and socio-emotional function (M = 26.5, SD = 11.5 versus M = 25.7, SD = 11.8), t (55) = 0.9, *p* = 0.360.

### Discussion

This randomized control trial investigated the efficacy of 20% albumin infusion in neonates with severe hyperbilirubinemia to enhance the binding of bilirubin in the plasma and consequently reduce TSB, the need for exchange transfusion, mortality during phototherapy, neurological impairment at discharge and improve developmental outcome at 12-months. The results reveal that albumin infusion lowers the TSB levels during treatment, but there were no significant changes in TSB levels and rate of change in TSB per hour between the albumin infusion arm and the control group. Additionally, no significant differences were found in other outcomes including the need for repeat phototherapy, exchange transfusion, inpatient mortality, and developmental outcomes at 12-months.

One of the reasons for the lack of effect of albumin infusion during phototherapy could be that the extraction of bilirubin from the tissues and its discharge into the blood depend on the phototherapy rather than additional albumin infused. These findings are similar to those reported by Hosono et al. and Tsao and Victor who reported no significant reduction in TSB levels between the 25% albumin infusion group and control group. The second reason for lack of additional effect of albumin infusion during phototherapy is that albumin may lead to an increase in oncotic pressure, expanding plasma volume hence delay the excretion of bilirubin from the body [20]. Third, the apparent ineffectiveness of albumin infusion may be due to oxidation of albumin during phototherapy. Oxidized albumin has a lesser affinity for bilirubin than albumin, thus promotes accumulation of free bilirubin in the body [19].

In a recent Cochrane meta-analysis evaluating the role of fluid supplementation in the treatment of NNJ, the authors reported that there were no differences between the treatment and control groups in the TSB levels 4 h after phototherapy [27]. However, several studies have reported the effectiveness of albumin infusion during exchange transfusion [21, 28, 29]. These studies have indicated that the effect of albumin infusion is useful in the clearance of bilirubin from the neonates’ tissues which may not be cleared by exchange transfusion.

## Limitations

There were a number of limitations to this trial. First, the trial was not a double-blind trial, although, the endpoints of bilirubin and the need for exchange transfusions are unlikely to be biased outcomes. Second, the plasma concentrations of unbound bilirubin were not consistently measured for participants in the treatment and control group, therefore, it is difficult to determine whether the infusion of albumin affected the unbound bilirubin or not. Additionally, the developmental assessments may not have detected more subtle impairments at 12 months of age that could impact functional abilities at a later age. Finally, the study had missing data on some participants hence they could not be included in this analysis. The missing data may potentially reduce the representativeness of the sample and introduced biasness in the estimations of the outcomes of this study.

## Supplementary information

**Additional file 1: Figure S1.** Enrolment, randomization, treatment, and evaluation. The figure is a CONSORT flow diagram that shows the progress through the different phases of the parallel randomised control trial. The information provided accounts for the participants enrolled, randomized, treated, and evaluated in the study.

## Data Availability

The data underlying the results are open access and available on the link 10.7910/DVN/TZ2RIH.

## Abbreviations

CGMRC: Center for Geographic Medicine Research-Coast
KEMRI: Kenya Medical Research Institute
TSB: total serum bilirubin
NNJ: neonatal jaundice
KCH: Kilifi County Hospital
IQR: interquartile range
