# Supplementary figures and images for: A randomized control trial of phototherapy and 20% albumin versus phototherapy and saline in Kilifi, Kenya
Source: BMC Res Notes. 2019 Sep 23;12:617. doi: 10.1186/s13104-019-4632-2 (PMC6757356; doi:10.1186/s13104-019-4632-2)

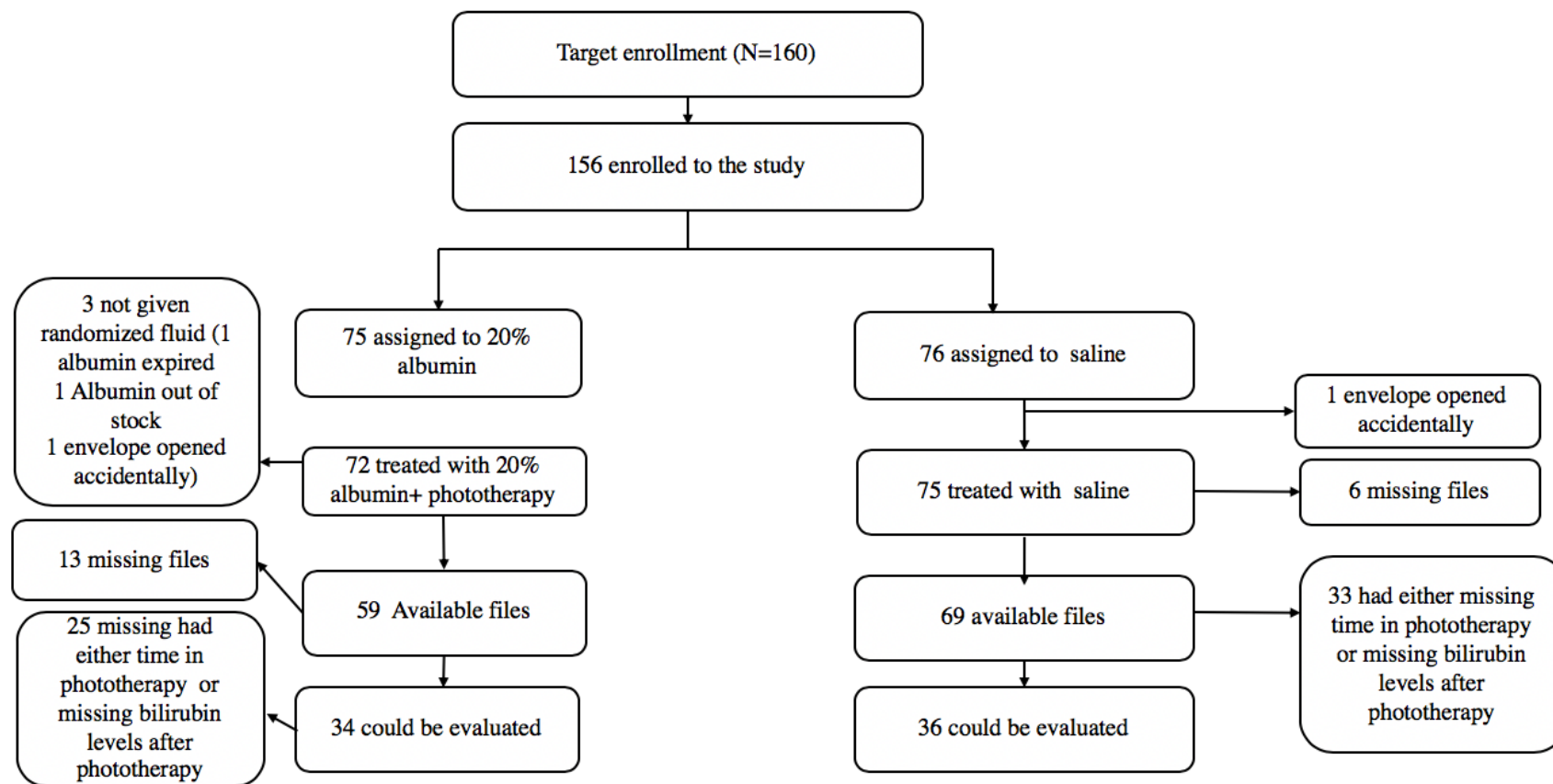

Figure S1. Enrolment, Randomization, Treatment, and Evaluation

Supplement: Supplementary file 1 — Additional file 1: Figure S1. Enrolment, randomization, treatment, and evaluation. The figure is a CONSORT flow diagram that shows the progress through the different phases of the parallel randomised control trial. The information provided accounts for the participants enrolled, randomized, treated, and evaluated in the study. [file 13104_2019_4632_MOESM1_ESM.pdf]
